# Age is not appropriate to be a mediating factor

**DOI:** 10.1097/JS9.0000000000001339

**Published:** 2024-03-12

**Authors:** Zheqi Fan, Houming Zhao, Jingcheng Zhou, Yiming Bi, Shuaifei Ji

**Affiliations:** Graduate School of PLA Medical College, Chinese PLA General Hospital and PLA Medical College, Beijing, People’s Republic of China

*Dear Editor,*

We were profoundly intrigued by the recent article titled ‘Glioblastoma patients’ survival and its relevant risk factors during the pre- and post-COVID-19 pandemic: Real-world cohort study in the USA and China’ by Qin *et al*.^1^ published in the *International Journal of Surgery*. The innovative approach of using the COVID-19 pandemic as a boundary to study the survival of glioblastoma patients is truly commendable.

The study employs mediation analysis to investigate the roles of age and bilateral tumor presence as risk factors influencing mortality. This approach offers new insights into the pivotal importance of a comprehensive treatment strategy to enhance survival rates for patients with glioblastoma multiforme (GBM), identifying age as the principal mediating variable. We deeply understand the significance of this study and believe that the research will help people view the impact of the COVID-19 pandemic from a new perspective.

However, we also believe there is an opportunity to further enhance the robustness of the study by reconsidering the use of age as a mediating variable in the mediation analysis. Age, being an objective variable, is not typically influenced by other factors than Relativity Theory time, making it less suitable as a mediating variable^2^. The authors hypothesized that the impact of comprehensive therapy on GBM survival might be partially or fully medicated by either age or tumor laterality. This hypothesis, while interesting, seems to contradict the basic principles of mediation analysis, as it implies that Comprehensive Therapy would influence age, which is unreasonable.

However, it needs to be explained that mediation analysis is still based on linear regression^3^. We do not deny the fact that the author found that age has statistical significance in the regression, but it should not be interpreted as a mediating factor. Age is more common in covariates.

We understand the authors’ intention to explore the role of age in GBM survival, and we propose considering the use of moderation analysis^4^. This would allow for an examination of whether age moderates the effect of the independent variable on the dependent variable. In the context of this article, the following can be described: Does age moderate the effect of Comprehensive Therapy on all-cause mortality? Providing a more nuanced understanding of the role of age in the treatment efficacy of Comprehensive Therapy. This can be visualized using a Johnson–Neyman plot.

We hope our suggestions will be seen as constructive feedback to enrich the conclusions of this already significant study. We anticipate with great interest the ongoing influence of this research as it brings to light fresh perspectives on the consequences of the COVID-19 pandemic. It is believed that, by considering our suggestions, the study could provide even more robust and nuanced insights into the complex interplay of factors affecting GBM survival.

## Ethical approval

Not applicable.

## Consent

Not applicable.

## Sources of funding

Not applicable.

## Author contribution

Z.F. and S.J.: study concept or design; Z.F., Y.B., and S.J.: writing the paper; Z.F., H.Z., and J.Z.: statistical support.

## Conflicts of interest disclosure

All authors declare that there are no conflicts of interest.

## Research registration unique identifying number (UIN)

Not applicable.

## Guarantor

The guarantor is the corresponding author.

## Data availability statement

Not applicable.

## Provenance and peer review

Not invited.
